# Public health round-up

**DOI:** 10.2471/BLT.18.010518

**Published:** 2018-05-01

**International Day of the Midwife devoted to quality of care**

A midwife speaks with a mother at the maternity ward at the Juba Teaching Hospital, Juba, South Sudan. The country has some of the highest maternal and infant mortality rates in the world. This year’s theme of International Day of the Midwife on 5 May is “midwives leading the way with quality care”. Read about the World Health Organization’s vision of quality midwifery care: bit.ly/2GOFM6K

**Figure:**
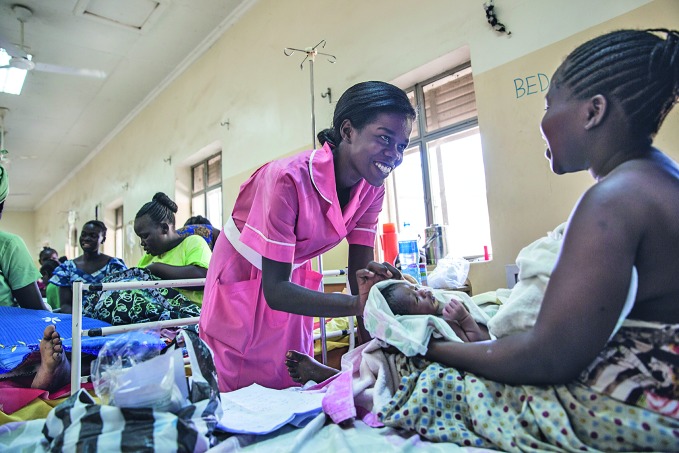
UNICEF/Mark Naftalin

## Yellow fever in Africa

A yellow fever vaccination campaign targeting nearly one billion people living in 27 African countries was launched last month.

The Eliminate yellow fever epidemics (EYE) strategy for the 27 countries was launched by Dr Tedros Adhanom Ghebreyesus, Director-General of the World Health Organization (WHO), and Professor Isaac Folorunso Adewole, Nigeria’s Minister of Health and partners at a meeting in Abuja, Nigeria. 

The acute viral haemorrhagic disease has emerged as a serious global public health threat following outbreaks in 2016 in densely populated cities in Angola and the Democratic Republic of Congo, that resulted in 400 deaths. 

The response to the two outbreaks exhausted global vaccine supplies and diverted public health resources from other health priorities. 

During the 10–12 April EYE strategy launch meeting in Africa, representatives from 14 African countries, WHO, the United Nations Children’s Fund, Gavi the Vaccine Alliance and other partners, developed a roadmap on how to roll-out the strategy at national level. 

These efforts follow the endorsement of the strategy by African health ministers at the 67th WHO regional committee meeting in September 2017.

The objectives of the strategy include protecting at-risk populations through preventive mass vaccination campaigns and routine immunization programmes, preventing international spread, and containing outbreaks rapidly. 

Developing strong surveillance, with robust laboratory networks, is key to these efforts.

The EYE strategy supports 40 countries (27 in Africa and 13 in the Americas) considered to be at high risk of yellow fever outbreaks and involves more than 50 partners. 

bit.ly/2JCXN9O

## Female genital mutilation clinical handbook 

Girls and women who have been subjected to female genital mutilation (FGM) rarely receive high quality health-care that meets their specific needs. This month WHO launched a new clinical handbook to help health-care workers provide such care.

The handbook, entitled *Care of girls and women living with female genital mutilation*, provides advice on how to care for and communicate with girls and women who have developed health complications due to FGM, as well as with their husbands, partners and relatives. 

The handbook is designed to support a wide range of health-care providers, including obstetricians, gynaecologists, surgeons, general practitioners, midwives, nurses and mental health professionals. 

It provides guidance on how health-care workers can provide quality health care to girls and women who have health problems due to FGM, including immediate and short-term urogynaecological and obstetric complications. 

Advice and clinical information for health workers is included, so that they can help affected girls and women make informed decisions about whether to undergo deinfibulation, a surgical procedure that can reverse a form of FGM known as infibulation.

One section of the handbook provides advice on how to support women who have mental health and sexual health complications caused by FGM. 

More than 200 million girls and women have undergone FGM in 30 countries in Africa, the Middle East and Asia, and the practice occurs in other countries as well.

bit.ly/2EAD7eU 

## Latent tuberculosis: guidelines 

WHO released consolidated guidelines on testing and treatment for tuberculosis infection, especially among groups who are particularly at risk, such as children and people living with human immunodeficiency virus (HIV). 

In* Latent tuberculosis infection: updated and consolidated guidelines for programmatic management,* WHO recommends action in three areas: identifying population groups that are at risk for latent tuberculosis infection, as well as, expanding testing and treatment options for these latent infections. 

WHO has also developed a mobile application to support programmatic management of latent tuberculosis infection. 

You can access the mobile application here: bit.ly/2ICjy8k.

## Fewer tuberculosis patients 

The number of new tuberculosis patients decreased at an average rate of 4.3% every year between 2007 and 2016 in the 53-country WHO European Region, according to a report by the European Centre for Disease Prevention and Control (ECDC) and the WHO Regional Office for Europe.

Despite this rapid decline, countries in the WHO Region are not on track to achieve the targets to end the tuberculosis epidemic. 

The Stop TB strategy sets the target of a 90% reduction in new cases by 2035. The target of sustainable development goal 3 is a reduction of 80% of cases by 2030, as compared with the baseline year of 2015. 

Low detection rates and inadequate treatment of multidrug-resistant tuberculosis (MDR–TB) are the main drivers of the tuberculosis epidemic in Europe, the report said. 

Data from the 2016 tuberculosis surveillance and monitoring report indicate that one in four people with MDR–TB in the WHO European Region does not receive a diagnosis. 

Diagnosis of MDR–TB patients increased from 33% in 2011 to 73% in 2016, but it remains to be seen whether the regional target of 85% will be achieved, as defined in the *Tuberculosis action plan for the WHO European Region 2016–2020*.

Treating patients with drug resistant forms of tuberculosis is another challenge: the observed increase in treatment success from 46% in 2013 to 55% in 2016 is still insufficient for countries in the European Region to reach the 75% target for 2020 they committed to in the action plan.

The spread of extensively drug-resistant tuberculosis (XDR-TB) is an additional threat to ending tuberculosis in the WHO European Region. With the rapid rollout of drug-susceptibility testing and improved surveillance, countries in the WHO European Region detected 5000 XDR–TB cases in 2016. On average only one in three patients with XDR–TB is cured. 

bit.ly/2EtZzGl

**Cover photo**

A man with his baby daughter in Ain Issa Camp in Raqqa governorate in the Syrian Arab Republic, where WHO is providing health services for camp residents through a health centre and a mobile health team.

**Figure:**
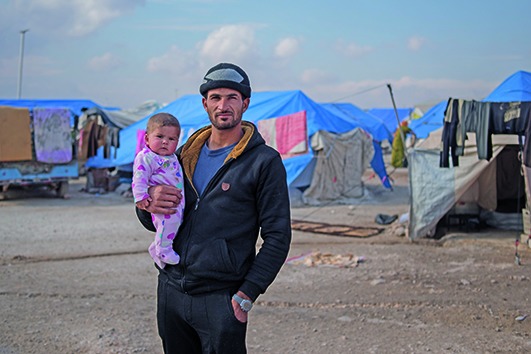
WHO/L. Mackenzie

## New testing guidelines

WHO released new interim guidance on the laboratory testing for Middle East respiratory syndrome coronavirus (MERS-CoV). 

The latest guidance, entitled *Laboratory testing for Middle East respiratory syndrome coronavirus interim guidance*, incorporates recent findings on the human viral infection and the immune response it elicits.

The new interim guidance – valid until December 2019 – covers indicators for testing, specimen collection and shipment, and laboratory biosafety measures for detecting MERS-CoV by polymerase chain reaction and sequencing. The guidance also provides details on available serological assays for MERS-CoV, and reporting of laboratory results. 

bit.ly/2qjkMi7

## Financing for noncommunicable diseases

About 400 delegates from countries, United Nations agencies, nongovernmental organizations, academia, philanthropy and business gathered last month in Copenhagen to explore new ways to fund the response to noncommunicable diseases (NCDs) in low- and middle-income countries.

Participants at the WHO Global Dialogue looked at examples of best practices for the alignment of public and private efforts to fund NCDs prevention and response, without incurring conflicts of interest.

As part of a youth initiative, 20 young people from 16 countries worked together to generate innovative ideas and solutions to help close the financing gap for NCDs.

The 9–11 April meeting was part of an informal process leading up to the United Nations General Assembly Third High-Level Meeting on the Prevention and Control of Noncommunicable Diseases in September 2018.

NCDs are responsible for 40 million deaths globally every year. Tackling NCDs is a global priority, but there is insufficient investment to achieve the United Nations Sustainable Development Goal target 3.4 of reducing premature deaths from NCDs by one third by 2030. 

bit.ly/2v6syAX

## Syrian conflict 

WHO called on the conflict parties in the Syrian Arab Republic to allow people to access health care, following reports of the suspected use of toxic chemicals in Douma city, East Ghouta.

In a statement issued last month, WHO expressed deep concern about reports of the use of chemical weapons. 

WHO and its partners in the health cluster are providing trauma care, medicines, supplies and personal protective equipment. The health cluster is the group of international medical and aid agencies that provide health care in emergencies. 

According to reports from WHO’s health cluster partners, during the shelling of Douma on 7 April, an estimated 500 patients presented to health facilities exhibiting signs and symptoms consistent with exposure to toxic chemicals. 

“WHO reminds parties to the conflict of their obligation to refrain from attacking medical facilities and personnel as per Security Council Resolution 2286 (2016),” the statement said. 

bit.ly/2qmKu5g

**Looking ahead**

21–26 May – Seventy-first World Health Assembly

31 May – World No Tobacco Day. Theme: Tobacco and heart disease.

26 September – UN General Assembly high-level meeting on ending tuberculosis, New York

9 October – WHO 2018 Symposium on health financing for UHC

30 October – 1 November – WHO Global Conference on Air Pollution and Health, Geneva

